# The Healing Process of Intracorporeally and In Situ Devitalized Distal Femur by Microwave in a Dog Model and Its Mechanical Properties *In Vitro*

**DOI:** 10.1371/journal.pone.0030505

**Published:** 2012-01-20

**Authors:** Zhenwei Ji, Yunlei Ma, Wei Li, Xiaoxiang Li, Guangyi Zhao, Zhe Yun, Jixian Qian, Qingyu Fan

**Affiliations:** Orthopedic Oncology Institute of Chinese PLA, Tangdu Hospital, The Fourth Military Medical University, Xi'an, Shaanxi, China; Pennington Biomedical Research Center, United States of America

## Abstract

**Background:**

Limb-salvage surgery has been well recognized as a standard treatment and alternative to amputation for patients with malignant bone tumors. Various limb-sparing techniques have been developed including tumor prosthesis, allograft, autograft and graft-prosthesis composite. However, each of these methods has short- and long-term disadvantages such as nonunion, mechanical failures and poor limb function. The technique of intracorporeal devitalization of tumor-bearing bone segment in situ by microwave-induced hyperthermia after separating it from surrounding normal tissues with a safe margin is a promising limb-salvage method, which may avoid some shortcomings encountered by the above-mentioned conventional techniques. The purpose of this study is to assess the healing process and revitalization potential of the devitalized bone segment by this method in a dog model. In addition, the immediate effect of microwave on the biomechanical properties of bone tissue was also explored in an *in vitro* experiment.

**Methods:**

We applied the microwave-induced hyperthermia to devitalize the distal femurs of dogs in situ. Using a monopole microwave antenna, we could produce a necrotic bone of nearly 20 mm in length in distal femur. Radiography, bone scintigraphy, microangiography, histology and functional evaluation were performed at 2 weeks and 1, 2, 3, 6, 9 and 12 months postoperatively to assess the healing process. In a biomechanical study, two kinds of bone specimens, 3 and 6 cm in length, were used for compression and three-point bending test respectively immediately after extracorporeally devitalized by microwave.

**Findings:**

An *in vivo* study showed that intracorporeally and in situ devitalized bone segment by microwave had great revitalization potential. An *in vitro* study revealed that the initial mechanical strength of the extracorporeally devitalized bone specimen may not be affected by microwave.

**Conclusion:**

Our results suggest that the intracorporeal microwave devitalization of tumor-bearing bone segment in situ may be a promising limb-salvage method.

## Introduction

Primary malignant bone tumors, mostly common in children and adolescents [1], [2], [3], threaten the survival and limb function of patients severely. In most cases, malignant bone tumors are located in the metaphyseal regions of long bones, especially around the knee joint [4]. Currently, limb-salvage surgery has been well recognized as a standard treatment and alternative to amputation for patients with malignant bone tumors [5], [6]. In general, limb-salvage surgery involves en bloc resection of bone tumors with a safe margin and reconstruction with different methods. To date, various techniques for reconstruction have been developed, including tumor prosthesis [7], allograft [8], [9], autograft [10] and graft-prosthesis composite [11], [12]. Particularly, the autograft refers to an extracorporeally devitalized tumor-bearing bone segment, usually by water bath heating [13], irradiation [14], autoclaving [15] or pasteurization [10].

When malignant bone tumors extend into the metaphyseal region of the knee joint, from an oncological point of view [16], [17], [18], [19], [20], it is safe to resect near the growth plate, thus the joint may be preserved. However, after the juxta-articular resection, if an endoprosthesis is used for reconstruction, it may be difficult for the remaining subchondral bone to hold the long intramedullary stem securely. In addition, if the intercalary bone graft is used, it may be hard to achieve rigid fixation with a steel plate due to the short residual subchondral bone [21]. Therefore, traditionally, in most cases, the resection usually involves the whole proximal or distal portion of the long bone including the articular surface. Under these conditions, most orthopaedic surgeons will have to adopt the arthroplasty [7], osteoarticular allograft [9], extracorporeally devitalized osteoarticular autograft [15], [22] or graft-prosthesis composite [11], [12], [23], [24] to reconstruct the bone defect. In this way, one or even two epiphyses would inevitably be lost or damaged.

As for the children and adolescents with malignant bone tumors, if the epiphysis is resected, a limb-length discrepancy and poor limb function could be anticipated. Furthermore, these established reconstruction methods also have other short- or long-term disadvantages [7], [8], [9], [25], [26], [27], including nonunion, deep infection, fracture and mechanical failures, which sometimes necessitate a second surgery in the future. Therefore, an alternative limb-salvage method, which can eradicate all tumor cells while avoiding those shortcomings mentioned above, is urgently needed.

Hyperthermia has now been used to treat many kinds of solid malignancies including bone tumors, especially in conjunction with radiotherapy or chemotherapy [28], [29], [30], [31], [32]. Image-guided microwave ablation, as a kind of hyperthermia therapy, has already been widely used to treat bone lesions of the spine, pelvis and long bones [33], [34], [35]. Presently, most of the studies and clinical reports have mainly focused on the minimally invasive treatment of small benign or metastatic bone tumors. Specifically, after inserting one or several antennas into the targeted bone lesions, the hyperthermia-induced ablation is performed. However, the studies on the ablation of larger and malignant bone tumors by microwave-induced hyperthermia are scarce. Instead of en bloc resection, our institution has opted to intracorporeally devitalize the tumor-bearing bone segment in situ with microwave after separating it from surrounding normal tissues with a safe margin [36], [37]. Postoperative follow-up indicated that the involved joints functioned well, as well as being stable and painless. Therefore, we speculate that, with this new limb-salvage method, the epiphyseal segment and articular cartilage could be preserved intact, and the above-mentioned complications could thus be avoided [8], [15], [21], [23], [38], [39].

For a wider clinical application of this technique, firstly we should have a thorough understanding about the revitalization potential of this kind of devitalized bone segment. However, little is currently known about its healing process and mechanical properties partially because the clinical specimens retrieved are rare and may have huge heterogeneity. The purpose of this study is to assess the healing process of intracorporeally and in situ devitalized bone segment in a dog model. In addition, as a pilot study, we also aim to explore the immediate effect of microwave on the biomechanical properties of bone tissue in an *in vitro* biomechanical test.

## Results

### Distribution of heat in distal femur

**Figure 1:**
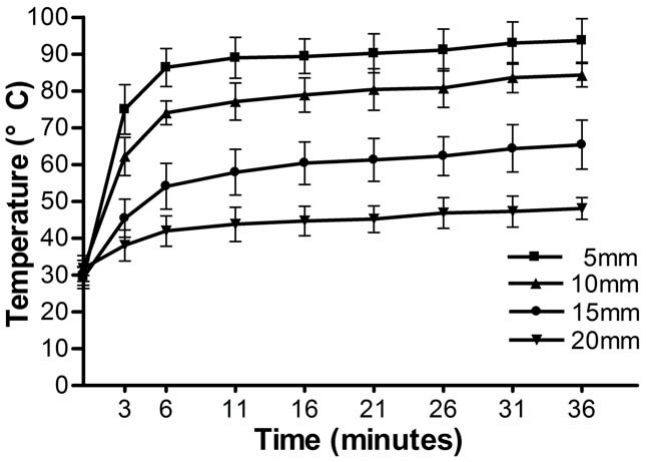
Temperature changes over time at 5, 10, 15 and 20 mm away from the antenna. The temperatures at drill holes 5 mm and 10 mm away from the microwave antenna rose to >70°C at the time interval of 6 minutes and continued to be between 70 and 100°C thereafter. The temperatures at drill holes 15 and 20 mm away from the microwave antenna were kept below 70°C all the time.

During intracorporeal devitalization, the temperatures of the targeted bone segment in distal femur (5, 10, 15 and 20 mm distal or proximal to the microwave antenna) were monitored and recorded every 3 or 5 minutes, and the temperature curves were shown in Figure 1. For drill holes 5 mm and 10 mm away form the microwave antenna, their temperatures had reached 86.4±5.1°C and 74±3.2°C respectively after 6 minutes of microwave-heating. While at the same time, the temperatures of drill holes 15 mm and 20 mm away from the antenna were only 54.1±6.2°C and 42±4.1°C respectively. Since then, approximately another 30 minutes of heating was continued, and the temperatures of all drill holes experienced a slight increase during this time.

### Complications and functional evaluation

24 of 25 dogs received intraoperative and in situ microwave devitalization, and one other dog did not receive any surgery or hyperthermia and served as normal control in microangiography. During the follow-up, 4 dogs (16.67%) developed fractures, all of which occurred between 1 and 3 months postoperatively. Autopsy revealed that all the fracture sites were located in the junction areas between heat-treated dead bone and surrounding normal bone proximally or distally. Another 3 dogs (12.5%) died of deep infection during 4 weeks postoperatively, which manifested as recurrent wound drainage and limb pitting edema. No other complications were observed. Full weight-bearing was allowed for all the microwave-treated dogs immediately after surgery, and limb function was evaluated before the dogs were sacrificed. According to the grading system [40], the outcome of limb function was excellent and good in 20 dogs (83.33%), and poor in 4 dogs (16.67%) that developed fracture during the follow-up. The knee joint of the treated limb, assessed by physical examination under general anesthesia, was stable, and passive full range of movement was evidenced in both knee joints.

### Radiological analysis

**Figure 2:**
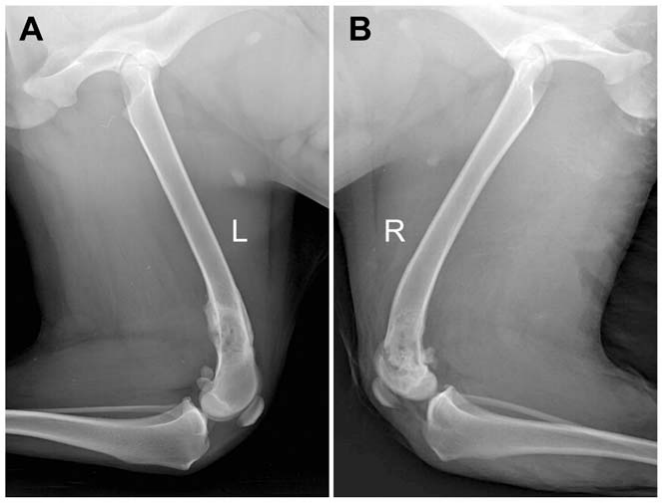
Radiological analysis of intracorporeally and in situ devitalized bone segment. (**A**) Plain radiograph showed periosteal callus formation covering the entire cortical surface of the devitalized bone segment at 3 months. (**B**) Partial sclerosis still remained within the microwave-treated bone segment at 12 months.

**Figure 3:**
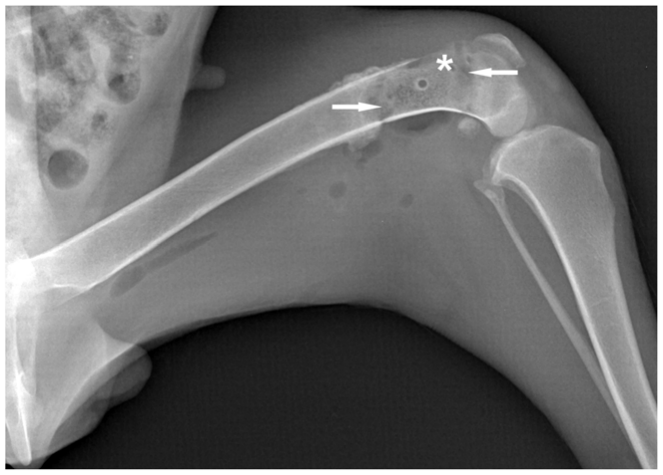
Radiological manifestation of bone resorption. Taken at 2 months after surgery, this radiograph showed a variant trend towards bone resorption. In this radiograph, the bone mineral density of the targeted bone segment was decreased, and in particular two crescent-shaped radiolucent lesions (arrows) were developed in its two ends between the dead bone segment and the normal bone tissues. In addition, focal cortical bone in the targeted bone segment became thinner or disappeared due to resorption (asterisk).

Radiological examinations were performed in 3 dogs at each time interval (2 weeks, 1, 2, 3, 6, 9 and 12 months) to assess the process of bone remodeling and to monitor potential complications. Periosteal callus formation was observed after 1–2 months, which was more frequently present at the proximal junction areas between the microwave-treated bone segment and the surrounding normal bone tissue. Periosteal callus extended gradually to the center of the devitalized bone segment and in some cases it could cover the entire cortical surface between 3 and 6 months (Figure 2A). The devitalized bone segment was characterized by an uneven sclerosis in radiograph. At the end of follow-up, the remodeling process of the microwave-treated bone segment was incomplete, with partial sclerosis still remaining in it (Figure 2B). In spite of the general trend towards healing, some variations were also observed during the follow-up. One of the most serious variations was the early and excessive bone resorption in the devitalized bone segment, which was shown in Figure 3. Furthermore, 4 fractures were also confirmed by radiography. No bone shortening, cartilage deterioration, subchondral bone collapse, or joint narrowing was evidenced in this study. In addition, a series of radiographs at all time points were provided in Figure S1.

### Bone scintigraphic results

**Figure 4:**
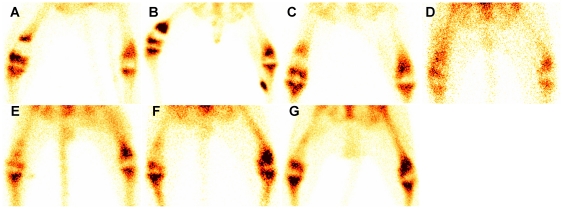
Bone scintigraphic analysis of devitalized bone segment at all time points. After intracorporeal devitalization of the targeted bone segment in situ by microwave, a series of bone scan images (**A–G**) were taken during the follow-up (2 weeks, 1, 2, 3, 6, 9 and 12 months). (**A**) In the beginning, the devitalized bone segment appeared as photon deficiency 2 weeks after surgery. (**B**) Focal hyperactivity of tracer uptake at the junction areas between microwave-treated bone segment and surrounding normal bone tissue was evident at 1 month. (**C–F**) The tracer uptake within the body of devitalized bone segment was detected between 1 and 2 months and increased gradually and diffusely thereafter. (**G**) Scintigraphic imaging showed that the intensity of radionuclide in the microwave-treated bone segment was still a little less than that in the contralateral limb at 12 months.

Technetium-99m-methylene diphosphonate (^99m^Tc-MDP) bone scintigraphy was performed in the same 3 dogs that received routine radiography at each time interval. In the initial period of follow-up, all the microwave-treated bone segments appeared as photon deficient areas (Figure 4A). Focal hyperactivity of tracer uptake at the junction areas was noted in all 3 dogs since 2 weeks postoperatively and lasted for 4–6 months (Figure 4A–4E). Focal uptake within the body of microwave-treated bone segment was detected between 1 and 2 months and increased gradually and diffusely thereafter (Figure 4B–4F). According to the evaluation method developed by Ehara [41], at the end of follow-up, all 3 dogs (grade -) showed evenly diffuse but less uptake in the microwave-treated bone segment than that in the contralateral side (Figure 4G). In addition, it was noted that the tracer uptake progressed from the two junction areas to the center of the microwave-treated bone segment gradually (Figure 4C–4E).

### Quantitative analysis of revascularization

**Figure 5:**
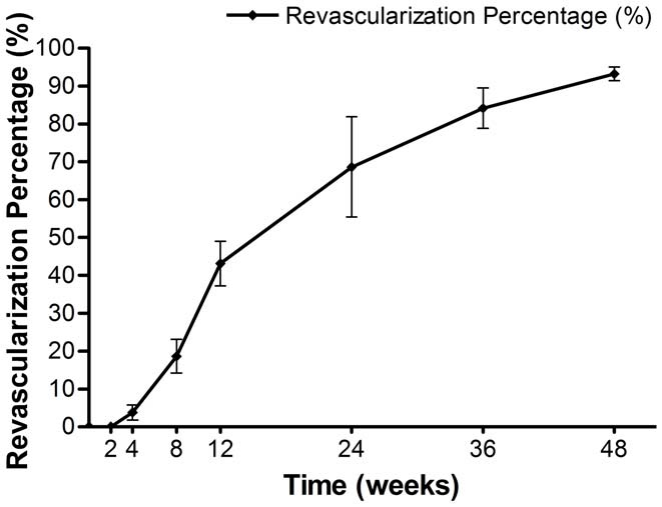
Revascularization percentage of devitalized bone segment at each time interval. At 2 weeks, there was no neovascularization in the cortical bone. At 1 month, the revascularization percentage was 3.78±1.99%, After then, the % revascularization increased rapidly to 18.65±4.44%, 43.12±5.89% and 68.66±13.20% at 2, 3 and 6 months respectively. While at 9 and 12 months, the percentage rose slowly up to 84.21±5.31% and 93.25±1.78% respectively.

Revascularization was evaluated at all time intervals by microangiography, and all the results of revascularization percentage (or % revascularization) were shown in Figure 5. At 2 weeks, there was no neovascularization in the cortical bone, the bone marrow cavity was filled with massive coagulative necrotic tissues and no recanalization was evidenced by Chinese ink. At 1 month, rare and scattered Haversian canals on the edge of cortical bone began to re-canalize, with the revascularization percentage being 3.78±1.99%. At this time point, in the bone marrow cavity, some areas adjacent to the endosteum were perfused with ink. After then, the revascularization percentage increased rapidly to 18.65±4.44%, 43.12±5.89% and 68.66±13.20% at 2, 3 and 6 months respectively. While at 9 and 12 months, the percentage rose slowly up to 84.21±5.31% and 93.25±1.78% respectively, with the latter being close to the normal level. At the same time, systemic microvascular network had been reconstructed in the bone marrow cavity.

### Histological analysis

**Figure 6:**
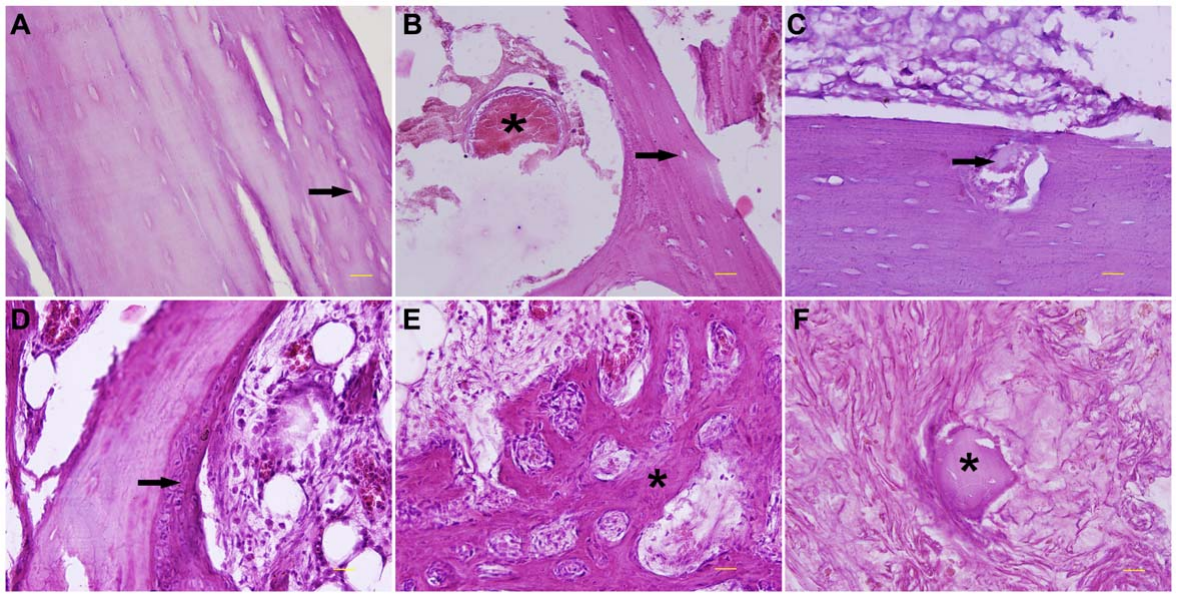
Histological analysis about the healing process of intracorporeally devitalized bone segment. (**A–F**) Haematoxylin and eosin staining. (**A**) Necrotic cortex with empty osseous lacunae (arrow) at 2 weeks. (**B**) Coagulative necrotic tissues (asterisk) and acellular trabeculae (arrow) in bone marrow cavity at 2 weeks. (**C**) Initial infiltration of fibrovascular tissues (arrow) into the dead bone at 2 months after surgery. (**D**) Partial substitution of dead trabeculae by new woven bone (arrow) at 3 months. (**E**) Newly formed trabecuale (asterisk) at 9 months. (**F**) Remnants of dead trabeculae (asterisk) surrounded by amounts of fibrovascular tissues at 12 months. Scale bars: 100 µm.

Histological changes with time were studied microscopically at all time points. At 2 and 4 weeks, the microscopical study revealed that the cortex at 10 mm distal to the microwave antenna was completely necrotic with empty osseous lacunae, while the architecture of the cortical bone remained intact (Figure 6A). The bone marrow cavity was filled with massive coagulative necrotic tissues and acellular trabeculae (Figure 6B), which were separated from the normal bone marrow cavity by reactive fibrovascular tissues. No cortical bone formation could be evidenced during the first month. At 2 months, fibrovascular tissues began to attach to the surface of the devitalized bone segment. At this time, some scattered areas in the cortical bone began to be infiltrated by these fibrovascular tissues (Figure 6C). In addition, amounts of fibrovascular tissues began to appear in the bone marrow cavity. Meanwhile, acelluar trabeculae were surrounded by these fibrovascular tissues, and newly formed woven bone was obvious along the dead trabecula's surface (Figure 6D). After then, the healing process progressed well, and amounts of newly formed cancellous bone were distributed among the bone marrow cavity (Figure 6E). However, there were still some acellular trabeculae scattered in the medullary cavity at the end of follow-up (Figure 6F), indicating the healing process had not concluded at 12 months postoperatively.

### Biomechanical results

As a pilot *in vitro* biomechanical study, the maximum load and stiffness values of bone specimens in compression and three-point bending test were measured and shown in Table 1, 2. As for the compression test, the bone specimens 3 cm in length were used. The maximum load and stiffness values of normal bone specimens were 9327.50±2446.64 N and 7044.82±1806.59 N/mm respectively, while those of extracorporeally microwave-treated bone specimens were 8855.00±1398.29 N and 8752.32±6254.32 N/mm respectively. The Student's *t*-test showed that there was no statistically significant difference between these two groups (*P*>0.05). For the bending test, the bone specimens 6 cm in length were used. The maximum load and stiffness values of normal control group were 1636.00±375.86 N and 718.96±355.01 N/mm respectively, while those of microwave-treated group were 1442.33±616.87 N and 691.30±401.62 N/mm respectively. There was also no statistically significant difference between these two groups (*P*>0.05).

**Table 1:** The maximum load values of bone specimens in the microwave-treated and control groups.

Group	Compression test (N)	Bending test (N)
Microwave-treated group	8855.00±1398.29	1442.33±616.87
Control group	9327.50±2446.64	1636.00±375.86
Statistical significance	p = 0.690 (Not Significant)	p = 0.526 (Not Significant)

**Table 2:** The stiffness values of bone specimens in the microwave-treated and control groups.

Group	Compression test (N/mm)	Bending test (N/mm)
Microwave-treated group	8752.32±6254.32	691.30±401.62
Control group	7044.82±1806.59	718.96±355.01
Statistical significance	p = 0.545 (Not Significant)	p = 0.902 (Not Significant)

## Discussion

Generally, as for the limb-salvage surgery, compared with conventional en bloc resection and the following reconstruction, the intracorporeal devitalization of tumor-bearing bone segment in situ by microwave-induced hyperthermia may have some additional advantages.

Firstly, when using allograft or extracorporeally devitalized autograft, one of the most important problems is bone union. Different from common fracture, the process of bone union between the viable host bone and dead allograft or autograft is very slow, which usually takes more than 1 year [15], [21], [23], [38], [42], [43], [44]. Furthermore, the incidence of bone nonunion is also considerably high, and a rate of nonunion between 9% and 33% has been reported [8], [15], [21], [23], [38], [39], [43]. Several factors may increase the incidence of bone nonunion including chemotherapy, radiotherapy and inadequate internal fixation [15], [23], [43], [45]. However, without en bloc resection, the intracorporeal and in situ devitalization can thus be free from this problem. Meanwhile, the healing process of this kind of devitalized bone segment might be quicker or promising for two reasons. The first one is that abundant blood supplies on its two sides can be preserved. The second one is that, without even the narrowest gap between graft and host bone as conventional limb-salvage methods have, this devitalized bone segment connects with the surrounding viable bone tissues structurally continuously and naturally.

Secondly, in most cases, conventional limb-salvage techniques usually involve en bloc resection of the whole proximal or distal portion of the long bone and reconstruction with arthroplasty, extracorporeally devitalized osteoarticular allograft or autograft or graft-prosthesis composite. Under these conditions, one or even two epiphyses and articular surfaces would inevitably be lost or damaged. In this way, limb-length discrepancy, poor limb function and other short- or long-term disadvantages could be anticipated. Currently, with progressive advances in biomedical engineering, microwave-induced hyperthermia can be precisely introduced into the deep-seated focal lesions such as those found in the skeletal system [33], [34], [35]. With targeted and controlled thermal treatment in situ, the objectives to eradicate all viable malignant cells with a safe margin and minimize the damage to surrounding normal tissues could be achieved simultaneously. Regarding the limb function in this study, 83.33% of dogs were considered acceptable (excellent or good). During the follow-up, all the dogs that healed uneventfully were found to have a stable joint and a passive full range of movement in the affected limbs. Furthermore, microscopical evaluation did not evidence any discernible injury to the articular cartilage. We therefore conclude that, without en bloc resection, the articular surface of the affected joint may be kept intact to a large extent by our method.

When considering microwave-induced hyperthermia as a treatment for human bone tumors, it must be ascertained that the tumor-bearing bone segment must be thoroughly devitalized, for definite killing of all tumor cells is a prerequisite for all curative methods [10], [15], [37], [46]. Previous animal experiment revealed that heating at temperatures >50°C for 20 minutes is enough to retard the growth of chondrosarcoma cells [47]. As for solid bone tumors, previous animal tests and clinical applications revealed that extracorporeal pasteurization (heating at 60–65°C for 30 minutes) is adequate to eradicate all tumor cells [10], [23], [24]. However, these data have been obtained in *in vitro* studies. It has been postulated that the thermal dose needed for definitely killing all bone tumor cells may be different *in vitro* and *in vivo*, since the heat dissipation by blood flow in cortical bone and marrow cavity may decrease the effect of heat treatment *in vivo* [48], [49]. Hence, to ensure a thorough intracorporeal devitalization, we prefer to adopt a temperature of >70°C for 30 minutes at the targeted bone segment. In this study, the combined results of a photon deficient area, no vascular perfusion and empty osseous lacunae in the targeted bone segment were sufficient to demonstrate the death of bone cells. Therefore, in this study, by a monopole microwave antenna, we could produce a necrotic area of nearly 20 mm in length in the distal femur.

In this study, histological examination revealed that newly formed bone tissue initially and predominantly occurred among the fibrovascular tissues, which migrated from the surrounding normal medullary cavity as reported by previous studies [22], [50]. This finding leads us to hypothesize that the first step for bone revitalization and new bone formation might be the migration of mesenchymal stem cells from the contiguous normal medullary cavity. Enneking et al. reported that the repair of a necrotic graft matrix was both external and internal, and the internal repair was confined to the ends and periphery of the cortex. They also thought that the internal repair penetrated slowly and incompletely, and furthermore, it seldom occurred in the later stage [43]. One reason for less internal repair might be the en bloc resection as in the conventional limb-salvage methods and the resultant interruption of cortex and Haversion canals, even if the closest contact between host bone and graft could be provided by the internal fixation. However, in our study, the microangiography and histological results revealed that the process of revascularization and healing developed rapidly from the two ends and periphery of the devitalized bone segment. These findings indicate that the internal repair may also play an important role in the healing process of the devitalized bone segment by our method. Therefore, we hypothesize that, instead of en bloc resection, the structural continuity and integrity of bone tissues provided specially by our method may contribute to the revascularization and remodeling.

We conducted a quantitative analysis of revascularization percentage to study the healing process. The results showed that the revascularization occurred at 1 month postoperatively and increased steadily thereafter. This change corresponded well with the bone scan results which showed the tracer uptake within the devitalized bone segment, starting from photon deficiency, experienced a gradual and diffuse increase during the follow-up. At 12 months postoperatively, the revascularization percentage reached nearly the normal level. However, at this time, the intensity of radionuclide in the microwave-treated bone segment was still a little less than normal. Furthermore, a small amount of necrotic trabeculae could still be found in the medullary cavity by histological examination. This discrepancy might be explained by the probable relationship between revascularization and revitalization or healing, that is to say, the former might occur earlier and be a precondition for the latter. Due to our medium period of follow-up, the complete healing of this kind of devitalized bone segment has not been confirmed this time. However, juxtaposed seams of woven bone among the fibrovascular tissues in marrow cavity might indicate a continuing healing potential. Therefore, a long-term follow-up study is necessary for a better understanding of the healing process of the devitalized bone segment treated by this method.

As for the biomechanical test, previous reports have indicated that the biomechanical properties of bone tissues devitalized by pasteurization (heating at 60–65°C for 30 minutes) could be kept intact to a large extent [51]. However, as in our limb-salvage method, to ensure a thorough intracorporeal devitalization, the temperature of 70–100°C for 30 minutes was adopted. Therefore, it is necessary to explore the effect of a higher temperature on the biomechanical qualities of bone tissue.

As a pilot study, we aimed to explore the immediate effect of this microwave-induced hyperthermia on bone's biomechanical properties. Specifically, immediately after extracorporeal devitalization (70–100°C for 30 minutes), the bone specimens were used for compression and three-point bending test. The bone strength against compression is mainly related to bone density, while that against bending is likely affected by bone collagen [51]. As shown in this study, there was no statistically significant difference between the microwave-treated group and the normal control. Therefore, our results indicated that the bone density and collagen of the devitalized bone specimens may be able to keep intact for a period of time at the very beginning, which corresponded well with other reports [51], [52]. However, considering the small sample size in this *in vitro* study, our results should be considered preliminary and further studies with ample numbers of subjects may strengthen these findings. In addition to the instant biomechanical changes *in vitro*, it is also very important to explore the time-dependent biomechanical changes of devitalized bone tissues *in vivo*. Considering its importance in predicting the possible complications, our future study is to investigate these dynamic biomechanical changes by testing ample subjects *in vivo* at every time point of the follow-up.

Fracture is one of the most serious complications for bone allografts and autografts, and the fracture incidences ranging from 10% to 45% have been reported previously [8], [10], [15], [21], [39], [42]. In this study, the fracture rate was 16.67%, which was comparable to the previous literature and was higher than anticipated. Fracture is thought to be attributed to the rapid and focal revascularization, which leads to bone resorption and the possible impairment of mechanical properties [22], [38], [42]. Additionally, unprotected weight-bearing and the drill holes for the microwave antenna and thermocouples might also increase the incidence of fracture. As shown in this study, bone scintigraphy and radiography may be valuable methods for monitoring the potential mechanical complications. Moreover, to protect the weakened bone and decrease the fracture rate during healing, rigid internal fixation and prolonged protected weight-bearing may be necessary. Furthermore, the rate of deep infection was 12.5% in this study, which was comparable to the previous results varying between 7% and 30% for comparable bone grafts [8], [10], [39], [42].

In general, we do not intend to suggest that the intracorporeal microwave devitalization of tumor-bearing bone segment in situ is the best limb-salvage method for all patients with malignant bone tumors. However, considering its unique advantages as mentioned above, it is thought to be a beneficial alternative in a certain number of appropriately selected patients, especially children and adolescents. Our pilot study revealed that this kind of devitalized bone segment may possess a certain healing potential. In addition, we found that the initial mechanical properties of bone specimens may not be affected by our method. Our study does, however, have a few limitations. The small sample size of dogs in each time interval group, the limited number of bone specimens in the biomechanical test and the medium-term follow-up might not enable us to reach any firm conclusions. However, as all the results in this study were consistent with each other, we feel confident in our findings. Meanwhile, our further studies in the future with ample numbers of subjects and long-term follow-up may strengthen these findings and confirm the superiority of this technique.

## Materials and Methods

### Ethics statement

All the experimental procedures involving animals were conducted under a protocol reviewed and approved by the Ethics Committee of Tangdu Hospital, Fourth Military Medical University (Permit Number: 2010016). All animal work was carried out in accordance with national and international guidelines to minimize suffering to animals.

### Surgical procedures and intracorporeal devitalization of distal femur in situ

25 adult beagle dogs (14 females and 11 males) weighing 16–25 kg were used in this study. One of the hindlimbs was randomly selected for surgical treatment, and the other one was left intact as a control in bone scintigraphy and radiography. Under general anesthesia, a 10 cm lateral incision approach was performed to expose the distal femur and knee joint. Periarticular muscles, ligaments and neurovascular bundles were separated from the distal femur and covered with wet dressings, which were moistened intermittently during heating. The posterior knee joint capsule and the soft-tissues attached to femoral medial and lateral condyles were kept intact. After drilling a coronal hole in the distal femur, which was 2–2.5 cm away from the proximal border of articular surface, the active tip (3 cm in length and 2.5 mm in diameter) of a monopole microwave antenna was introduced into it. In addition, an array of parallel drill holes (5, 10, 15 and 20 mm away from the antenna) were made proximally or distally to the antenna, into which the thermocouples were inserted for monitoring the temperature. A diagram of location distribution of the antenna and thermocouples was shown in Figure S2. The antenna was connected to a 2450 MHz microwave generator with power output ranging from 0–100 W. Localized heating was performed, and the temperature was monitored continuously and recorded at 0, 3 and 6 minutes and every 5 minutes thereafter. The microwave generator was controlled manually to maintain the antenna temperature below 100°C. The whole heating time was approximately 36 minutes. After heating, the necrotic periosteum and eschar were cleared completely. The surgical site was thoroughly rinsed with physiological saline. The soft tissues and skin were sutured in three layers. Antibiotics (cefazolin 25 mg/kg) were administered intramuscularly every 2 hours during surgery and for 5–7 days postoperatively (cefazolin 25 mg/kg, twice daily). No cast immobilization was applied. The dogs were allowed to walk unrestrictedly immediately after surgery.

21 out of 25 dogs were divided into seven groups randomly (3 dogs in each group), with one group for each time interval of the follow-up (2 weeks, 1, 2, 3, 6, 9 and 12 months). Another 3 dogs were chosen randomly for radiography and ^99m^Tc-MDP bone scintigraphy at every time interval. The last one dog served as a normal control in microangiography, which did not receive any surgery or microwave treatment. During the follow-up, the dogs were assessed for limb function and healing process macroscopically and microscopically. Before sacrificed, one dog in each group and the above-mentioned normal dog were chosen randomly for microangiography.

### Functional and radiological evaluation

During the follow-up, the limb function and the complications of deep infection and fracture were examined in all the microwave-treated dogs. Limb function during walk was evaluated according to the following grading system: excellent (slight or no lameness), good (mild lameness), fair (evident lameness) and poor (severe lameness or no use of the limb) [40]. The range of movement and joint stability of the knee were also examined when the dogs were under general anesthesia. Radiological examination was performed in 3 dogs at each time interval. In addition, the dogs that might have developed fractures were also confirmed by radiography. Radiographs were obtained and evaluated for fracture, resorption, subchondral bone, and joint narrowing according to the International Society of Limb Salvage (ISOLOS) radiological implants evaluation system [53].

### Technetium-99m-methylene diphosphonate (^99m^Tc-MDP) bone scintigraphy

To assess the revascularization and metabolic activity of the intracorporeally devitalized bone segment, bone scintigraphy was performed in the same 3 dogs that received radiography at every time point. Imaging began 2–3 h following the intravenous injection of ^99m^Tc-MDP (18.5 MBq/kg). A local bone scan was performed and static image including the two hindlimbs was obtained. For scintigraphic evaluation of the bone segment sterilized in situ, the radionuclide uptake is graded as (−) if the uptake is less than the normal contralateral bone, (0) if it is similar to the normal bone and (+) if it is more than that of the normal bone as suggested by Ehara [41].

### Microangiography

One dog in each time interval group and one normal control dog (8 dogs in all) were chosen randomly for quantitative analysis of revascularization percentage with microangiography. Under general anesthesia, the femoral artery and vein of the affected limb were exposed and catheterized with appropriate cannulae after ligation. Normal saline containing heparin (12,500 IU/L) was injected through the artery cannula until the ejected venous blood became clear. Then 1 L of filtered perfusate containing 50% black Chinese ink, 40% low molecular weight dextran, and 10% buffered formalin was administered until the skin of the limb stained black uniformly. After sacrificed by air injection intravenously, the dog was stored at 4°C for 2 h, and then the affected femur was retrieved. The transversal specimen, which was 5 mm in thickness and distal to the drill hole for microwave antenna, and the longitudinal semi-cylindrical specimen, which was 10 mm in length and proximal to the antenna hole, were cut by an oscillating saw. The specimens were embedded in polymethylmethacrylate (PMMA) and cut into sections 100 µm in thickness. These sections were studied by light microscopy. Next, using image analysis software (Image-Pro Plus, version 5.1), we quantitatively measured the revascularization percentage in the microwave-treated bone segment. The revascularization percentage is defined as the ratio of area of blood vessels in the heat-treated bone to that in untreated control bone [13].

### Histological Examination

One dog in each time interval group (7 dogs in all) was chosen randomly for histological examination. After sacrificed, the microwave-treated bone segments were retrieved, cut into transversal and longitudinal specimens and fixed with buffered formalin. After decalcification, the specimens were embedded in paraffin and cut into sections 10 µm in thickness. The sections were stained with hematoxylin and eosin (HE staining) and studied by light microscopy to assess the revitalization process of devitalized bone segment.

### Biomechanical Examination

After sacrifice at each time point, the normal contralateral limbs of dogs were collected and stored in a −80°C refrigerator. After defrosting, these femurs were made into two kinds of bone specimens, 30 mm and 60 mm in length respectively, with the number of each kind being 12.

#### Compression test

Twelve 30 mm-bone specimens were divided into two groups randomly, with group A for microwave treatment and group B for normal control. The 6 bone specimens in group A were heated by inserting a microwave antenna into the bone marrow cavity. The heating temperature and duration adopted *in vitro* were the same as that used in the above-mentioned *in vivo* experiment. Next, the bone specimen was placed between two parallel round platens or rollers (12 cm in diameter), with the specimen's long axis matching the compression axis. The compressive strength was applied at a speed of 1 mm per minute, and the load deflection curve was recorded simultaneously. Then the maximum load (N) and stiffness (N/mm) values were derived according to this curve. The remaining 30 mm-bone samples in the two groups were tested in the same way. All the maximum load (N) and stiffness (N/mm) values in these two groups were analyzed by Student's *t*-test.

#### Three-point bending test

Twelve 60 mm-bone specimens were divided into two groups randomly, with group C for microwave treatment and group D for normal control. The 6 bone specimens in group C were heated in the same way as in group A. After then, all the specimens in group C and D were used for the bending test. Both 5 mm ends of the 60 mm-bone specimen were placed on two supports 50 mm apart, and then the bending force was applied by a blunt-rounded-edge (1.25 cm in width) at the midpoint of the specimen at a speed of 1 mm per minute. The load deflection curve was recorded, and the maximum load (N) and stiffness (N/mm) values were derived. The data of three-point bending test in these two groups were analyzed by Student's *t*-test.

### Statistical analysis

The data were reported as mean value ± standard deviation. Statistical differences between groups were analyzed by two-tailed Student's *t*-test. Differences between groups were regarded as statistically significant if *P* was less than 0.05. All statistical analysis was performed with statistical analysis software (SPSS, version 17.0).

## Supporting Information

Figure S1 **Radiographs of divitalized bone segment by microwave at all time points.** After intracorporeal and in situ devitalization of the targeted bone segment in distal femur, a series of plain radiographs (**A–G**) were taken during the follow-up (2 weeks, 1, 2, 3, 6, 9 and 12 months). (**H**) The contralateral limb was provided as a normal control.
(TIF)

Figure S2 **Diagram of positions of the microwave antenna and thermocouples.** In the distal femur, a microwave antenna was inserted into the bone coronally through a drill hole (short arrow) which was 2–2.5 cm away from the proximal border of articular surface (asterisk). After then, an array of parallel drill holes (long arrow, 5, 10, 15 and 20 mm away from the antenna) were made proximally or distally to the antenna, into which the thermocouples were inserted for monitoring the temperature.
(TIF)
