# Supplementary figures and images for: The Healing Process of Intracorporeally and In Situ Devitalized Distal Femur by Microwave in a Dog Model and Its Mechanical Properties In Vitro
Source: PLoS One. 2012 Jan 20;7(1):e30505. doi: 10.1371/journal.pone.0030505 (PMC3262834; doi:10.1371/journal.pone.0030505)

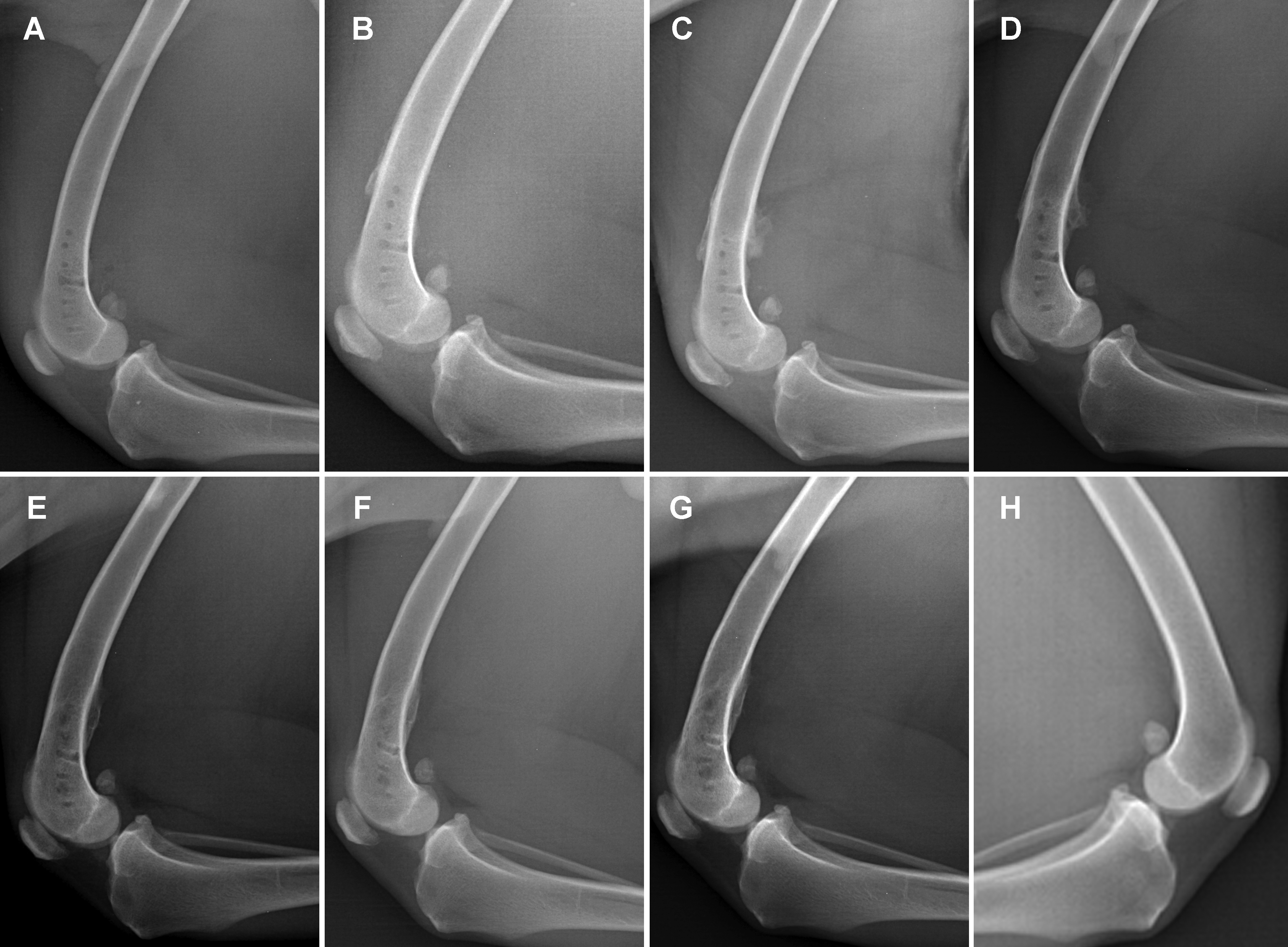

Supplement: Figure S1 — Radiographs of divitalized bone segment by microwave at all time points. After intracorporeal and in situ devitalization of the targeted bone segment in distal femur, a series of plain radiographs (A–G) were taken during the follow-up (2 weeks, 1, 2, 3, 6, 9 and 12 months). (H) The contralateral limb was provided as a normal control. (TIF) [file pone.0030505.s001.tif]

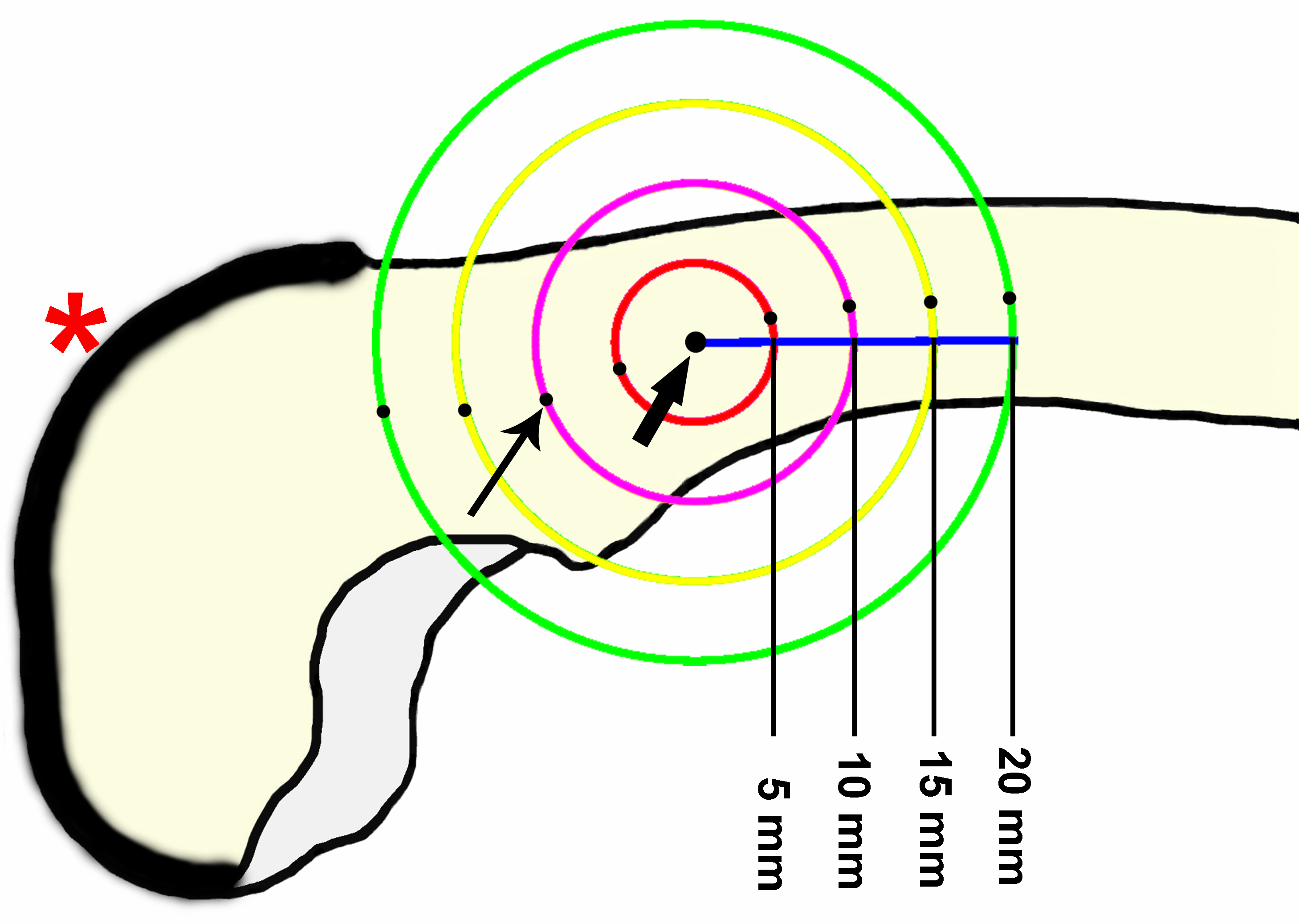

Supplement: Figure S2 — Diagram of positions of the microwave antenna and thermocouples. In the distal femur, a microwave antenna was inserted into the bone coronally through a drill hole (short arrow) which was 2–2.5 cm away from the proximal border of articular surface (asterisk). After then, an array of parallel drill holes (long arrow, 5, 10, 15 and 20 mm away from the antenna) were made proximally or distally to the antenna, into which the thermocouples were inserted for monitoring the temperature. (TIF) [file pone.0030505.s002.tif]
